# Considerations for colorectal cancer surgery in Greece during Covid-19 pandemic

**DOI:** 10.11604/pamj.supp.2020.35.2.23287

**Published:** 2020-05-13

**Authors:** Athanasios Syllaios, Spyridon Davakis, Nikolaos Garmpis, Konstantinos Stephanos Mylonas, Adamantios Michalinos, Ioannis Karavokyros

**Affiliations:** 1 First Department of Surgery, Laiko General Hospital, National and Kapodistrian University of Athens, Athens, Greece; 2 Second Department of Propedeutic Surgery, Laiko General Hospital, National and Kapodistrian University of Athens, Athens, Greece

**Keywords:** Colorectal cancer, Covid-19, pandemic, Greece

## To the editors of the Pan African Medical Journal

A novel coronavirus disease (COVID-19) was initially reported in December 2019 in China and was rapidly declared a pandemic by World Health Organization challenging healthcare systems at a global level. Many elective surgeries were postponed worldwide in an effort to minimize spread of disease as well as to conserve resources. With the ongoing need for emergency and elective colorectal cancer surgery several issues and concerns raise from this situation while management of those patients globally including Greece has changed [1]. In Greece, due to reduction in number of operating rooms and in an effort to conserve medical staff and ventilators, delays in elective cancer surgery occur. Patients requiring surgery are mandatorily being screened for COVID-19 infection and most of them have to remain in hospital while waiting approximately 1 week for an elective colon cancer surgery due to surgery cancellations. Although uncommon, some patients with advanced colorectal tumors may undergo neoadjuvant chemotherapy and/or radiotherapy due to the fear being operated during the pandemic and may receive longer schemes of neoadjuvant chemotherapy. Considering the fact that digestive tumors are indolent, there are cases where asymptomatic patients became symptomatic (pain, rupture, bleeding, obstruction) especially in colon and rectal cancer and required emergent surgery. In our hospital, during the pandemic in April 2020, a higher percentage of colorectal cancer patients (approximately 30%) were admitted and operated on emergently than before the pandemic (approximately 10%). Due to the risk of aerolised dissemination of the Covid-19, minimally-invasive colectomies and low anterior resections were drastically reduced and most of the colorectal cancer surgeries are performed with open laparotomy. Finally, there is limited availability of staging advanced imaging and elective colonoscopies and access to them has become extremely difficult.

Based on the Italian experience, within colorectal emergency surgery, only life-threatening emergencies should be treated (i.e. intestinal perforation, obstruction and bleeding), in order to spare crucial resources for the management of the pandemic and an open approach is recommended [1]. Yu et al. proposed suggestions that are different from daily practice as regards operation for colorectal cancer under the outbreak of corona virus disease based on the Chinese corona virus experience [2]. To minimize caregiver exposure, endoscopic procedures should not be performed in the office due to the lack of appropriate environment and personal protective equipment, but in endoscopy suites that include a negative pressure endoscopy room. Also, the use of an active smoke evacuator connected to a proper filter has been recommended for laparoscopic or robotic colorectal cancer procedures during the COVID-19 pandemic [3].

## Conclusion

COVID-19 pandemic is seriously affecting the structure of healthcare systems globally. Many interventions will be needed to deal with the new scenario after the crisis has been controlled. Colorectal cancer surgery is arguably life-threatening if not conducted within a “reasonable” time-frame and there is an urgent need to re-group and rethink the way the surgical care is being offered in this subgroup of patients during the COVID-19 pandemic.

## Competing interests

The author declares no competing interests.
